# ICT-Enabled Mobile Work: Challenges and Opportunities for Occupational Health and Safety Systems

**DOI:** 10.3390/ijerph17207498

**Published:** 2020-10-15

**Authors:** Swantje Robelski, Sabine Sommer

**Affiliations:** Federal Institute for Occupational Safety and Health, Nöldnerstraße 40-42, 10317 Berlin, Germany; sommer.sabine@baua.bund.de

**Keywords:** flexible work arrangements, institutional health and safety systems, mobile work, occupational health and safety, OHS

## Abstract

The development and dissemination of new technologies has brought forward a rise in flexible work arrangements, such as mobile work. In the literature, mobile work has mostly been discussed from a microergonomic perspective, considering detachment, stress, strain, and life-domain balance. However, the macroergonomic or institutional perspectives have often been neglected, although for occupational health and safety (OHS) management, as well as occupational health and safety systems, many questions remain unanswered. Therefore, in the present paper, information and communication technologies (ICT)-enabled mobile work is described taking into account institutional and regulative, as well as company-related, requirements. As the literature-based analysis was able to show, existing regulations cover many aspects of mobile work arrangements but also offer starting points for a more concrete protection of mobile workers. Furthermore, there are challenges regarding the enforcement of regulation. In this regard, new technologies might offer the chance to improve the interactions between institutional and company-related occupational health and safety systems. Additionally, 278 co-funded research projects in Germany were categorized, yielding 18 projects on new ways of work, of which another eleven projects addressed different aspects of mobile work. The project analysis revealed that current research focuses on tools and strategies for designing communication and cooperation. In conclusion, the examination of research trends can be used to generate new knowledge for better OHS management and effective OHS systems.

## 1. Introduction

New developments in sensor technology, robotics, and the rise of cyber-physical systems have been termed Industry 4.0. For industry 4.0, the central technology is not the computer but the internet. Along with worldwide networking across company or country borders, the digitization of production is taking on a new quality that triggers and enables new forms of work organization [1]. For many employees, this means being able to work more detached from physical workplaces. Especially jobs in the service industry or highly qualified jobs are being transferred off the worksite, an evolving trend [2] that very recently has been reinforced by the spread of the Coronavirus. About 70% of HR managers from almost 500 companies indicated in a survey that their “office workers” worked at least partially from home [3]. According to a survey initiated by Eurofound, 36.9% of respondents from Germany reported that they started to work from home as a result of the situation, while only 16.7% of respondents indicated to have already worked from home before the outbreak [4].

As will be shown in the course of the study, research on flexible work arrangements identified both advantages, as well as disadvantages, on the individual, social, and organizational levels [5]. However, several shortcomings with regards to occupational health and safety (OHS) structures and instruments have been identified. On a company level, questions that need to be answered revolve around the organization of flexible work arrangements, as well as their risk assessments. On an institutional level, there is concern on how regulations can be enforced. In this regard, some authors have observed an increasing invisibility of occupational health and safety in the digitized world of work, because occupational health and safety practitioners, as well as institutional agents, are increasingly losing access to employees who were previously situated at the employer’s premises [6]. In the same vein, it was pointed out that fractured labor markets (including home-based work or multi-employer worksites) constitute difficulties in ensuring standards, allocating responsibility, and enforcing laws [7].

The challenge of flexible work arrangements is furthermore aggravated by a lack of adequate instruments and understanding of psychosocial problems, because the traditional perspective of OHS in terms of physically measurable hazards and technical solutions is still prevailing [8]. As an example, the latest European Survey of Enterprises on New and Emerging Risks (ESENER) found that, across all sectors, procedures for stress prevention were available for only about 30% of the workplaces [9]. In a German sample, only 21% of companies conducted a psychosocial risk assessment [10].

For the purpose of this paper, the focus rests on flexible work arrangements and, specifically, mobile work. Mobile work can be understood as work that is tied neither to the workplace at home nor to the workplace at the company worksite, for it is strongly relying on (mobile) information and communication technologies (ICT). If mobile work takes place at home, it can be differentiated between telework and home office. The first is characterized by a fixed workplace in the home of the employee, which is (often) equipped with the support of the employer. Another main characteristic of telework can be seen in a contractual agreement with regards to its duration and extent between employer and employee [11]. A home office or work from home (WFH) is practiced in a less-regulated fashion. In the course of the article, we will refer to mobile work or remote working as the more generic terms and refer specifically to WFH or telework if necessary.

According to the European Working Conditions Survey (EWCS), most employees (about 70%) report to have a single regular workplace. Employees with multiple worksites (30%) indicated to be working at the client’s/customer’s or patient’s place, at home, or even underway. Public places such as coffee shops were less frequently visited for work purposes [12]. Similarly, in a representative survey on working times, 75% of German employees stated that their workplace was not mobile [13]. With regards to mobile workers, data from the last EWCS showed that traditional occupations such as in construction or agriculture still account for the majority of mobile workers [14]. Focusing specifically on telework, the latest Bundesinstitut für Berufsbildung/ Bundesanstalt für Arbeitsschutz und Arbeitsmedizin (BIBB/BAuA) employee survey by the Federal Institute for Vocational Education and Training (Bundesinstitut für Berufsbildung, BIBB) and the Federal Institute for Occupational Safety and Health (Bundesanstalt für Arbeitsschutz und Arbeitsmedizin, BAuA) that is representative for Germany found that about 12% of employees work from home as teleworkers [15]. However, it can be assumed that the proportion increases if noncontractually regulated mobile work was also included. On a European level, 13% of the company’s representatives indicated that some of their employees work from home in ESENER 2, thus showing comparable results for EU28 [16]. As the data from BIBB/BAuA indicated, the proportion of telework has increased over the course of the last years [15]. It is suggested that this increase cannot be attributed solely to demographic changes or a shift towards knowledge work [17].

Drawing upon ICT use outside of the employer’s premises, the EWCS differentiates teleworkers (as those working mainly from home) from mobile workers (working at multiple sites). According to the EWCS data, “around 9% of workers in the EU use ICT outside of the employer’s premises: 2% telework mainly from home and 7% are exclusively ICT-mobile workers” [12] (p. 86). ICT mobile workers were found often in the financial service sector, as well as at higher occupational levels (e.g., managers) [12]. Again, this result is mirrored by data from the German working time survey. About 3% of respondents indicated their work was not bound to a specific place. The majority of this subsample was employees in knowledge-intensive occupations such as computer sciences [13]. With regards to knowledge workers, there does not seem to be a great escape into public places. The employer’s premise remains the preferred workplace, and the extension of office boundaries mostly encompasses the home [14].

As the current numbers show, the single workplace remains the prevailing form of workplace organization. However, there is a growing part of employees reporting to work mobile based on the use of ICT. Hence, in the present study, we will be answering the question if OHS systems in their current form of regulation are able to protect workers sufficiently from hazards introduced by mobile work. Furthermore, we will present a new approach to identifying what knowledge is available and needed to provide an effective means of protection. Therefore, the paper firstly describes the development of a framework for OHS, as well as the methodological approach used for the project analysis. Following this, the current regulations regarding mobile work from an institutional and company-level point of view are described based on the framework, and the results of the qualitative analysis of research projects conducted in Germany are presented in order to deduce approaches for the design of mobile work with regards to health and safety. Both aspects are jointly discussed, and recommendations for the design of OSH systems are proposed.

## 2. Methods

The present study’s aim was twofold. On the one hand, a framework for describing OHS on an institutional and company level was developed and used for the analysis of mobile work. On the other hand, a project analysis was conducted for identifying the current research endeavors regarding the design of mobile work. Therefore, different methodological approaches were followed.

### 2.1. Development of a Framework for OHS on an Institutional and Company Level

**Figure 1:**
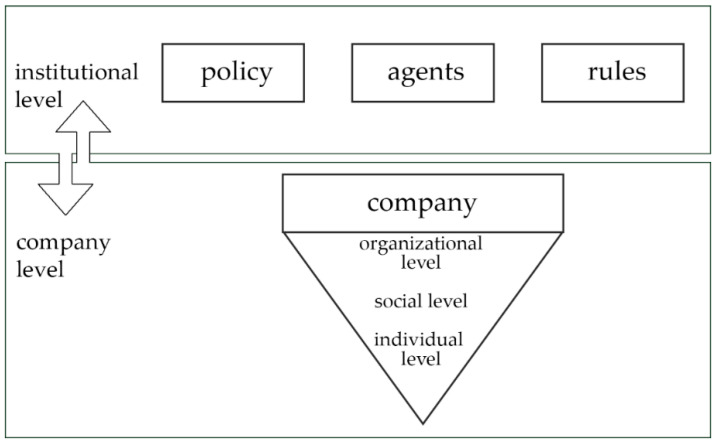
Framework for occupational health and safety (OHS) systems based on the institutional and organizational levels.

According to the International Labour Organization (ILO) convention C187 for a promotional framework for occupational safety and health, a national OHS system is based on a national policy, national system, and a national program, as well as a national safety culture [18]. The framework presented herein relies on an adaption of the ILO’s promotional framework and consists of an institutional level composed of policy and strategy, agents who are responsible for enforcing a special set of rules, and regulations referring to occupational health and safety, as can be seen in Figure 1.

The second level of the framework refers to the company, which is understood in terms of the organizational, social, and individual levels. In this regard, the organizational level represents factors such as technical equipment, company-wide rules, processes, and structures playing an important role for OHS. The social level refers to the interactions and communications between employees and includes teamwork or leadership. On the individual level, OHS-related aspects such as demands and resources, skills, and competences are taken into account.

A literature review and a regulations analysis regarding mobile work were used to fill the framework with content and describe the different points of view embedded in the institutional and company-level OHS systems.

### 2.2. Project Analysis

For the project analysis, we focused on a nationally funded line of research that was initiated by the German Ministry for Education and Research in 2015. Since then, the funding program called “innovations for production, service industries and future work” continuously funds research on production systems, service industries, and the future of work. Within the last five years, several calls on specific topics have been issued. The whole program is supposed to be stocked with a funding volume of almost a billion Euro [19]. For all calls, independent experts evaluated project suggestions according to predefined specifications, such as novelty, consortium, and expected impact [20]. For projects to be deemed fundable, research questions have to be proposed by a consortium composed of research institutions and small- and medium-sized enterprises (SMEs) or large company partners. In that way, practice-oriented research and long-term interests in the results are supposed to be secured. Both completed and running projects were taken into consideration, with the latest projects having started in June 2020.

As of 15.07.2020, 278 projects were manually transferred from an online project database into an Excel sheet with information concerning acronyms, short descriptions, weblinks, evaluation of the relevance, relation to OHS, and entry date. In the first step, based on the projects’ short descriptions, they were categorized regarding their relevance into either irrelevant or relevant. The latter included projects whose contents were directly answering questions of institutional and occupational OHS or whose proposed ideas could be transferred to OHS.

In the course of the analysis, an inductively developed classification was applied, in which the projects’ contents and relation to OHS was either classified as:

- New technologies: comprising the description of newly developed technologies like production techniques and artificial intelligence.
- Occupational health and safety management: referring explicitly to possibilities for organizing and managing occupational safety and health, e.g., instructions.
- Forms of employment and flexible work arrangements: solutions and approaches on how to deal with flexibility.
- Business models and management: descriptions of business models or modes of operation in businesses enabled by new technologies (e.g., platforms and horizontal integrations of manufacturers and customers).

Another researcher categorized 50 randomly chosen projects according to their relevance. Cohen’s kappa was used a measurement on the interrater reliability [21]. The kappa was quite low (κ = 0.22), respectively “fair”, according to the classification of Landis and Koch [22]. However, as can be seen in the cross-table, which is provided in the Supplementary Materials (Table S1), Rater 1 (S.R.) was stricter with regards to categorizing projects as relevant or irrelevant. Raters talked about differences, and Rater 1 adapted the evaluation strategy for further categorization accordingly.

## 3. Analysis of OSH-Related Responses to Mobile Work on an Institutional and Company Level in the German Context Using the Framework

As was described in Section 2.1, the current framework for describing OHS is composed of two levels. On the one hand, there is the institutional level. On the other hand, there is the company level. Both levels and their specifics will be outlined in the next section.

### 3.1. Institutional Level

The institutional level refers to policy and strategy, agents, and rules. All three elements are described with regards to their requirements for mobile work.

#### 3.1.1. Policy and Strategy

The general understanding of OHS refers to historically grown employment relationships characterized by employees working full-time at the employer’s premises. However, the German coalition agreement for the 19th term of parliament stated the intention to undertake research regarding flexible work arrangements, as well as to focus on occupational safety and health in the context of digitization [23]. A new dynamic could be observed with the outbreak of the Coronavirus. A right to work from home was discussed controversially [24]. While employee representatives appreciate the initiative and refer to their own proposition [25], employer representatives decline the notion. On a European Level, the Directive 2019/1158 on work-life balance for parents and carers [26] can be expected to influence national policy.

Although occupational health and safety in Germany develops positively with regards to occupational accidents [27], there is a call for new guiding principles regarding the visibility of digitized work, as well as support for OHS agents [6].

#### 3.1.2. Agents

In our framework, agents refer to institutions and stakeholders of the OHS system. In Germany, the OHS system is based on two pillars. While the federal state is responsible for OHS laws and acts at the national level, the enforcement of these laws is borne by the states (Länder and Bundesländer). Additionally, there are social accident insurance institutions (Berufsgenossenschaften and Unfallkassen) whose main objective is the prevention of occupational accidents and occupational diseases, as well as work-related health risks. The social accident insurance institutions—organized according to industries—enact and implement accident prevention regulations (Unfallverhütungsvorschriften). Another task of accident insurance institutions is rehabilitation and compensation, as well as advising employees and employers on work-related risks [28].

Flexible work arrangements and, especially, flexible workplaces are challenging for national OHS agents, since access to employees is aggravated. As the results from a qualitative study show, in traditional work arrangements, institutional OHS agents found their way into businesses, even if it was challenging. With increasingly flexible work arrangements, the problem of access is amplified, and knowledge about labor practices gets lost. OHS actors such as inspectors need to find new ways to get access to employees [6]. During inspections, OHS inspectors should be informed about the number of employees working from workplaces other than the business premises. It is doubted that current modes of inspection are able to provide the necessary level of protection, since new risks often cannot be detected by the examination of records or inspection of single workplaces [29]. As most OHS inspectors have acquired additional skills on psychosocial demands and risk assessment [30], an expansion of training in order to capture flexible work arrangements and to gain a shared understanding seems promising.

For accident insurance institutions, insurance legal questions arise if employees work temporarily or permanently off the employer’s premises. In the case of accidents, it has to be established if the accident was task-related or had other reasons. There is a problem of demarcation, since occupational and personal risks overlap [31]. Furthermore, mobile work and increased ICT use have been identified as a priority, and new prevention concepts on telework and working from home are being developed [32,33].

#### 3.1.3. Rules

The framework furthermore consists of rules and regulations as the means used by institutions to achieve their objectives (e.g., accident prevention and safe workplaces). Generally, it can be stated that the German Occupational Safety and Health Act [34], as well as the German Working Hours Act [35], regulate the rights and obligations referring to occupational health and safety at work, as well as working times. These acts are applicable for all workplaces and provide a protective foundation [36].

However, with the presence of a telework agreement, regulations that are more specific become applicable, such as the German Workplace Ordinance (ArbStättV), which requires employers to ensure certain standards of workplace design in terms of working environment and equipment, including work with visual displays [37]. Mobile work (without a contractual agreement) is explicitly excluded from the regulation in the assumption that it would only occur occasionally and, thus, would not need specific OHS standards. However, as was shown with regards to dissemination, mobile work in general and WFH are gaining importance. Therefore, new approaches to regulation could furthermore increase the level of protection.

### 3.2. Company Level

The German Occupational Safety and Health Act requires employers to provide either unharmful working conditions or the means to reduce possible hazards for life and health [34]. This national implementation of the Council Directive 89/391/EEC installs occupational risk assessments as a pivotal instrument for employers to identify hazards and implement appropriate preventive means [38]. This risk assessment has to take into account working tasks, technical factors, the working environment, work organization, and social relations [34]. However, several studies were able to show that risk assessment is conducted in only about half of German companies, with an immense structural bias in the form of small- and medium-sized enterprises (SME) lacking to provide risk assessments [10,39]. Furthermore, according to ESENER 2, of those establishments carrying out risk assessments and having reported that employees work from home, only 29% of respondents indicated that risk assessments would also cover the workplaces at home [16].

Another important instrument is the directive on machinery, which obligates manufacturers of machines to provide information on the machine’s safety, so that employers can ensure that only safe machines are used at the workplace [40].

OHS management at the company level furthermore obligates employers to appoint OHS practitioners, as well as occupational medicines, for their employees, according to the Occupational Safety Act (Arbeitssicherheitsgesetz, ASiG). Both have to cooperate with employee representatives on OHS-relevant decisions [41].

Several ordinances and technical standards furthermore specify German national law on occupational health and safety. In addition to European or national legislation, company and employee representatives can lay down company agreements. In cases where the employer is part of an employer’s association and employees are represented by a labor union, collective agreements might be used to regulate aspects such as working times or telework.

#### 3.2.1. Organizational Level

On an organizational level, employers are responsible for their employees’ health and safety. As described in Section 3.2, they are required to identify threats to life and safety and install respective means if necessary. The occupational risk assessment is a pivotal OHS instrument. In this regard, the introduction of mobile work should be covered by risk assessments. As the Joint German Occupational Safety and Health Strategy (Gemeinsame Deutsche Arbeitsschutzstrategie, GDA) proposed, updates of the risk assessment should be made as soon as there are changes in the task or working environment [42]. For the case of flexible work arrangements, updating or repeating the risk assessment several months after mobile work has been introduced could be advantageous, since employees would have the chance to get to know the specific demands. Furthermore, the German Occupational Health and Safety Act demands instruction at several instances [34]. The introduction of mobile work can be seen as such a situation.

To prepare employees for the specifics of mobile work, training courses might be suggested. In a Canadian study, most home-based workers indicated that they had not received any training but appreciated such an offer in order to facilitate the adaption to telework [43].

Employee representatives and employers can negotiate employment agreements in order to meet company-specific demands that are not addressed by general regulations. In this regard, an analysis of 31 company agreements was able to demonstrate the broad spectrum of contents and solutions, although important aspects such as leadership and corporate culture were not found to be covered thus far [44]. Additionally, a question that is gaining importance refers to the availability and impact of employee representatives. With an increasingly dispersed workforce, companies have to find ways and means to enable representation.

In summary, the successful implementation of mobile work requires an awareness of the legislation, as well as careful preparation in terms of structures, process, and those involved [45].

#### 3.2.2. Social Level

On a social level, work processes should be organized in a manner that enable mobile work. Time schedules have to be adapted to provide time for (virtual) meetings, calls, and undisturbed working times. Furthermore, it must be taken into consideration that team processes might be completely altered if there are less face-to-face interactions. Team members report a decrease in performance, either if they are working from home themselves or if a coworker is working from home [46]. From a managerial perspective, the labor productivity of the team decreases the more time team members spend working from home (with no team member working from home as the reference) [46]. However, a meta-analysis did not find negative effects on relationships with supervisors and coworkers [47]. Nevertheless, supervisors and team leaders are challenged to adapt their leadership styles and to incorporate OHS into the distributed work. A recent study was able to show that a leader’s exhibition of health and safety-specific leadership was related to positive outcomes in distributed workers, such as feeling part of the organization or being more proactive in safety matters [48].

#### 3.2.3. Individual Level

Telework and ICT-based mobile (TICTM) work arrangements offer more autonomy compared to single workplaces outside the home [47,49]. Although remote or flexible workers reported higher organizational commitments, enthusiasm, overall job satisfaction, and work-life balance [17,47,50,51], main office workers felt more included than home-based workers, workers in satellite offices, and client-based workers [52]. At the same time, remote workers were shown to spend more effort than fixed-place workers in terms of working hours, work intensity, and voluntary efforts [17]. The increase in work intensification has also been termed the autonomy paradox [49]. Generally, increased demands regarding self-organization and decreased detachment were observed [53]. Negative effects on well-being through interruptions could also be demonstrated [51]. TICTM workers were more likely to report stress and psychosocial problems, thus indicating risks for mental and physical well-being [49]. An emerging risk can be seen in a new form of (virtual) presenteeism characterized by mobile workers continuing to work despite sickness [49], which is also indicated by a low number of sick days among mobile workers [53].

Technology characteristics (such as IT presenteeism and the pace of IT change), as well as the intensity of telework, in terms of days per week, were identified as influencing factors for the occurrence of stressors (e.g., work overload and invasion of privacy) and susceptibility for technostress [54].

The presented results on flexible work arrangements at the company level can only be seen as an exemplary account of changes and challenges related to mobile work. Depending on company-specific arrangements, different outcomes on the psychosocial level might occur. Therefore, implementing mobile work should not be looked at in isolation but in the context of organizational processes, working tasks, equipping, and individual needs.

## 4. Project Analysis of Current Research on Mobile Work

The project analysis was conducted with the aim of deriving design approaches for mobile work from the current state of the research.

**Figure 2:**
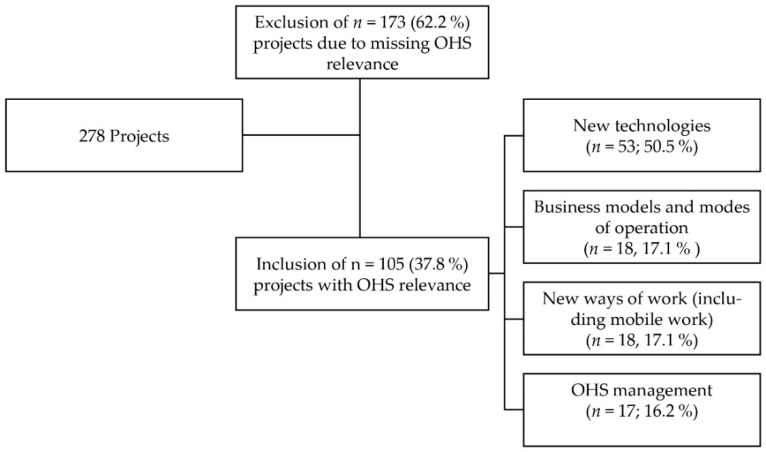
Flow chart of the selection process; *n* = 105 projects were included in the analysis.

The project analysis included 278 projects regarding “innovations for production, service industries, and future work”. As described in Section 2.2, a first evaluation was based on the relevance and transferability to occupational health and safety, leading to the exclusion of 173 projects whose content primarily did not touch OHS-related aspects. As Figure 2 shows, the remaining 105 projects were broadly categorized, with projects describing the development or application of new technologies constituting the main proportion (45.5%). Projects concerning business models and modes of operation, new ways of work, or OHS management each accounted for about one-sixth.

There were 18 projects dealing with new ways of work (*n* = 18) on which a qualitative analysis based on accessible content was conducted in order to further describe the research activities. In the course of the analysis, another seven projects were excluded due to the examination of different work arrangements (e.g., specializing on production systems and crowd work) and digitization in general without focusing on mobile aspects or recent project starts, so that the results cannot be expected as of yet. A complete list of the projects classified as dealing with new ways of work is provided in the Supplementary Materials (Table S2). Since the line of research analyzed funded projects with the participation of companies, the company level of the framework presented in Figure 1 was used to describe eleven projects whose contents included aspects of mobile work. However, the projects might address several company levels at the same time, and the classification is not distinct, as can also be seen in Table 1. Therefore, the projects dealing with aspects of mobile work are classified according the predominant level.

As described in the framework above, the **organizational level** refers to aspects of work organizations, structures, and OHS elements. The EdA project worked on questions of empowerment in a digitized world of work shaped by increasing agility. An important aspect taken into consideration referred to worker participation and the understanding that employee representation can be a stabilizing factor for increasingly flexible work [55]. Although ReProNa mainly deals with project work, the tool for reflexive learning that is being developed can also be seen as a way to establish sustainable structures for organizational learning. As stated in the projects’ objectives, lessons learned are supposed to be used to counteract the obstacles of project work, such as problematic team structures, resource planning, or the dissatisfaction of employees [56]. In a similar vein, knowledge management in (virtual) teams can be a challenge. Therefore, WiViTe developed an IT framework and analyzed the organizational perspective [57]. In SANDRA, a smart assistant is supposed to be responsible for e-mail and phone call management on mobile phones in order to delay or block messages and adapt availability settings in leisure time by using natural language processing [58]. Thus, the smart assistant system is an organizational response to deal with increasing boundarylessness. In prentimo, the risk assessment methods were adapted to mobile work, thus representing the organizational level by referring to the employers’ responsibility to adequately assess threats to employees’ health and safety. Success factors for the risk assessments of mobile work included, among others, early information of employees and the applicability on mobile devices [59]. Furthermore, in diGAP, it was pointed out that framework conditions regarding structures, roles, equipment, and qualification must be met for agile and trans-local teams. Company agreements are another instrument that might support healthy agile work [60].

The **social level** includes interactions between employees, as well as aspects of teamwork and leadership. ReProNa, WiViTe, and SANDRA can also be understood in terms of the social level, since they provide important information for teamwork if all or some team members are working mobile. Focusing on agile work, the project diGAP recommended for health-oriented agile project work to strengthen the self-organization of the team, e.g., by providing adequate resources [60]. diGAP also addressed the challenge of using agile methods not as a locally organized (office-based) form of work but, rather, in trans-local teams. It was found that, consequently, both process- and subject-relatedness between team members decreased. Proposed strategies to deal with this challenge referred to socializing events, joint kick-offs, distributed roles, and the usage of chat groups. Especially the latter might be seen as a way of trying to reproduce a local room digitally [61]. Comparably, case studies conducted within the project CollaboTeam showed that digital, collaborative applications have a potential for reducing the corrosive effects of distance. Enterprise Social Media seems promising for low-threshold communication—provided by the firm, the usage of nonprofessional and potentially insecure products can be avoided [62]. However, as proposed in vLead, a team mental model on media—including a shared understanding of media characteristics, media use, and the respective rules of conduct—might support task-, team-, and time-related team mental models [63]. Similarly, praevierull recommended the requirement to establish rules on communication and media use. Regarding mobile work and distributed teams, an important part of leaders’ roles will be to guide the connection and interaction between team members and experts, as well as to provide an overview and process-related knowledge [64,65]. Leadership plays an important role for mobile work and also relates to the social level. In this regard, the objective of the project teamIn was to use artificial intelligence (AI) as a means to support leaders in organizational tasks such as personnel planning in order to enable more time for leadership functions such as motivating [66]. The development of qualification modules for conversational leadership in socio-digital systems was targeted by eLLa4.0 [67].

On the **individual level,** demands and resources, skills, and competencies related to mobile work were also addressed in the projects. In this regard, praevierull pointed out that, with the increase in ICT-based communication, the extraction of relevant information amongst an abundance of information should be recognized as an increasing demand for leaders and employees. Therefore, competences regarding technology use and social-communicative competences are gaining importance [64]. As time schedules in mobile work arrangements become less predictable, SANDRA can also be seen as a project referring to the individual level of the company, since the assistance system contributes to individual availability management. The project prentimo analyzed mobile work in various facets (including service technicians, as well as IT consultants), including an individual point of view. Examining different occupations and forms of mobility, certain characteristics were found to be similarly demanding, including time pressure, limited social support, and recreational possibilities across the respective occupations [59]. In conclusion, the projects analyzed addressed different aspects of mobile work. The social level prevailed but was intricately linked to the organizational and individual levels.

## 5. Discussion

In the following section, the strengths and limitations of the study will be presented. Furthermore, the results will be discussed against the background of the current literature, and proposals for future research will be derived.

### 5.1. Strengths and Limitations

In the ongoing discussion concerning mobile work arrangements, the well-structured approach presented within the paper proved to be useful. All relevant stakeholders and their positions were considered using the framework, thus showing the complex dependencies and relations between them. It was shown that OHS systems need to be prepared for flexible work arrangements, especially when used at the broad scale, such as recently practiced due to the Coronavirus. By analyzing the current status quo, we were able to point out strengths and shortcomings in the current legislation. However, aspects such as information security and working hours could only be touched upon.

The project analysis was an innovative approach for establishing an overview on the research landscape. It can provide useful information in combination with other methods, such as literature reviews. Since there might be some form of time lag regarding scientifically published results, relying on information from the project database allowed a timely overview of the current research. The examined line of research is interdisciplinary and encompasses strong efforts to include psychosocial, ethical, or legal issues into the analysis. Additionally, as the analyzed line of research explicitly required collaborative projects between researchers and companies, the projects can be seen as positive examples for research to practice, as well as application-related solutions. Drawing from these trends in the current research, propositions for future system designs can be made. Due to the qualitative approach, research projects dealing with different aspects of mobile work were considered, even if they were not explicitly termed that way (e.g., knowledge management, project work, and distributed teams).

However, there are several limitations that have to be taken into consideration. The present paper focuses on the German OHS system and legislation. Furthermore, the projects analyzed were mostly financed and conducted in Germany. Thus, the generalizability of the results is constrained.

Since only a specific line of funding was examined, other important research might have been missed. Despite our efforts for the transparent selection and categorization of projects, different assignments and a certain ambiguity in the project categorization cannot be ruled out.

Another limitation concerning project analysis refers to the different stages of the project’s progress. As described in Section 2.2, parts of the research have already been completed, while other undertakings have just started. Therefore, assertions concerning the projects’ success in accomplishing their objectives cannot be made. Prior to the funding decision, project outlines have been evaluated by experts, and a general applicability seems to be given, though.

Despite some limitations in terms of transferability to different national contexts, the results can be discussed, and recommendations for the design and accessibility of mobile work can be made.

### 5.2. Discussion of Results

The analysis presented within the paper was able to show that, on an institutional level, the current legislative framework offers a foundation for OHS in mobile work that is enforceable in principle. However, as the world of work changes, the traditional understanding of workplaces must be reassessed. As of today, contractual agreements play an important role for making flexible work arrangements such as mobile work accessible to representation, protection, and specific forms of regulatory supervision. In this regard, it is imaginable to extend the workplace an ordinance’s scope of application on mobile work or to introduce standards for work with mobile display units [31]. Generally, it can be assumed that an expansion of regulation could contribute to a higher level of protection [49]. A current EU-tender concerning the Workplace and Display Screen Equipment Directives (EMPL/LUX/2020/OP/0006) is supposed to elicit options for updating both regulations [68] and might be seen as a starting point for further political activities.

With regards to institutional agents, increasing the invisibility of the workforce and decreasing possibilities for access aggravate enforcement. This problem is exacerbated by a continuous downscaling of OHS inspectors during the last years [69]. Additionally, thus far, the use of technical equipment in inspectorates is limited [70]. However, reports of positive experiences concerning e-government functions or the provision of risk assessment tools have been described [71]. Increased ICT use or the use of new technologies such as AI have also been suggested to be related to potential benefits, such as savings and the increased effectiveness of inspections and enforcements, although implementation costs should not be underrated [71,72]. In the project analysis, there were no projects focusing specifically on institutional OHS, which can be explained by the funding program chosen for the analysis. Nevertheless, technological approaches such as platform-enabled interconnections might offer starting points for collaboration and communication between institutional OHS agents and companies, although rigorous requirements on data protection must be fulfilled. In a similar vein, using digital tools such as augmented or virtual reality for OHS planning [73] could also facilitate cooperation between organizational and institutional agents.

The organizational level plays an important role for the implementation of mobile work, as the employer has responsibility for his employees’ safety and health. In this regard, conducting adequate risk assessments is a major obligation, which is highlighted by the results on psychosocial demands related to mobile work. The project analysis was able to show that research on risk assessment methods specifically designed for mobile work is being conducted. However, there is a general lack of compliance related to the implementation of risk assessments in companies [10]. A possible reason why companies refrain from conducting risk assessments is a rather physical understanding of the risk factors and the alleged absence of such [74]. However, there is a gap between the use of digital technologies and the consideration of their possible health-related impacts on workers, as findings from a survey on new and emerging risks in enterprises indicates [9]. In the light of cautious findings on a positive relation between technostress and decreased mental health [75,76], psychosocial risk assessments are an important means of occupational prevention. Therefore, raising awareness for the impact of work equipment and work organization on OHS in a psychosocial and physical understanding already is, and will be remaining, a challenge.

Furthermore, knowledge management plays an important role on a company level. While the projects examined put emphasis on a content- and task-related focus, OHS-related aspects could also be addressed, such as instructions and support for the implementation of mobile work or workstation design. As a study on telework indicated, almost 60% of participants did not receive advice or training on the installation of home-based equipment [43]. In this regard, digital tools might prove to be helpful in order to implement a participatory approach to ergonomics. Digital tools for cooperation and availability management were also addressed in the projects. Especially the latter is gaining importance, since mobile workers (telework and home office) were found to indicate longer working hours and more overtime [15,49]. Compulsory rest periods of eleven hours between the end of a working day and the start of new one are more often neglected than indicated by employees not working from home [15].

Mobile work heavily relies on the use of ICT, and mobile workers are challenged to increasingly organize themselves. Thus, tools for collaboration, as well as adequate ICT equipment, are essential. However, outside of telework agreements, employees often have to rely on their private ICT, which is a grey area not only in terms of product safety but, also, in terms of information security. The latter might be limited if employees are forced to fall back onto less professional options. The company-driven provision of ICT for mobile work should offer advantages regarding product safety and information security. In sum, interventions, such as the implementation of new technologies or new ways of work organization, cannot only be considered in isolation but cover the entire company [77] and should, therefore, be carefully monitored.

Based on the discussions and limitations encountered in the presented study, an agenda for future research can be proposed. On the one hand, there is a need for more longitudinal research on working conditions of ICT-enabled mobile work outside the traditional company workplace, as well as regarding the interplay of organizational, social, and individual requirements for occupational safety and health. Regarding the institutional level, to this date, the use of new technologies is limited in German labor inspectorates. Therefore, future research should address labor inspector’s demands and needs regarding technologies supporting their tasks (e.g., using mobile devices, AI, and digital access of company data). At the same time, inspector’s technology acceptance and technostress should be investigated, as both play an important role for successful implementation on an institutional level. Furthermore, efforts for improving the interplay between organizational and institutional agents (e.g., using platforms), as well as their backflow of information, should be made in order to gain a better understanding of the task and function design.

## 6. Conclusions

The current COVID-19 pandemic signifies a break of serious consequence, as the numbers in mobile work—specifically telework and home office—skyrocketed. Up to now, the new normal remains unclear, but it can be assumed that there is no going back regarding traditional attendance at the workplace [3]. However, experiences of the current situation are helpful in order to define the processes and structures necessary for OHS-appropriate mobile work arrangements in the long term. In these regards, the present study can be seen as a starting point for a continuative discussion of institutional and organizational OHS systems and their readiness for a changing world of work that should be further accompanied by research activities.

As the analysis presented herein showed, the current German OHS system is designed to be generally valid and can therefore cover many aspects of mobile work. However, a higher level of protection could be achieved by explicitly considering mobile work in the regulations. From the point of view of the institutional OHS system, mobile work poses a particular challenge for policy and agents. Answers to the question of what an appropriate enforcement of rules looks like must be found. From the point of view of the companies, however, clearer guidelines can be helpful. On the company level, new technologies and flexible work arrangements inherit a new quality of demands for employees, as well as challenges for corporate occupational health and safety actors, but they also hold the potential to be turned into new solutions. All in all, several challenges for occupational and institutional OHS systems to be prepared for a more mobile and digitized world of work remain. Nevertheless, using the project analysis, we were able to show that the current research offers a lot of potential for solutions and can provide new knowledge for better occupational health safety management. Promisingly, various approaches are referring to technical solutions, as well as organizational, social, or individual considerations. New technologies have enabled employees to step out of the boundaries of the traditional office. However, now is the time to make use of new technologies to make this step a health-oriented one.

## Figures and Tables

**Table 1:** Projects on mobile work referring to the company level.

	Company Level	Organizational Level	Social Level	Individual Level
Project	Collabo Team		✓	
	diGAP	✓	✓	
	EDA	✓		
	eLLa4.0		✓	
	Praeviernull		✓	✓
	prentimo	✓		✓
	ReProNa	✓	✓	
	SANDRA	✓	✓	✓
	teamIN		✓	
	vLead		✓	
	WiViTe	✓	✓	
		6	9	3

## Supplementary Materials

The following are available online at https://www.mdpi.com/1660-4601/17/20/7498/s1: Table S1: A cross-table for the interrater analysis. Table S2: Research projects referring to new ways of work within the project line analyzed.
