# Supplementary material for: ICT-Enabled Mobile Work: Challenges and Opportunities for Occupational Health and Safety Systems
Source: Int J Environ Res Public Health. 2020 Oct 15;17(20):7498. doi: 10.3390/ijerph17207498 (PMC7602556; doi:10.3390/ijerph17207498)
Supplement: Supplementary file 1 [file ijerph-17-07498-s001.pdf]

Supplementary

Table S1. Cross-table for calculation of Cohen's Kappa.

		Rater 1		Sum	%
		0	1		
**Rater 2**	0	12	1	13	26
	1	22	15	37	74
	**Sum**	34	16		
	**%**	68	32		

Table S2. Research projects referring to new ways of work within the project line analyzed.

Acronym	Title [1]	Research Goals [3]	Weblink	Duration	Mobile Work (yes/no)
Cloud-Crowd	Cloud and Crowd as a challenge—sustainable design of new organizational concepts for services	• Development of cloud and crowd based organizational concepts for the design, provision and distribution of services.	http://cloud-und-crowd.de/	12/15–06/19	n
CollaboTeam	Collaborative team-and project-work	• development of exemplary, holistic design concepts focusing on the connection between collaboration software and work design<br>• Testing of the concepts in four application areas (software development, IT services, mechanical engineering, and logistics)	http://www.collaboteam.de	02/17–10/20 [1]	y
COVID19-LL	COVID-19 Lessons Learned	• systematic and supra-regional collection and supply of Best Practice examples for new ways of work resulting from the COVID-19-related measures taken in different industries	https://www.mw.tum.de/lfe/forschung/projekte/covid-19-ll/	06/20–05/21	n
diGAP	Good agile project work	• examining components of agile project work that prove to be particularly contradictory and endanger the success of the project<br>• development and testing of digital working models and training concepts for good agile project work, which will be integrated into company processes at the partner companies<br>• these models are supposed to support agile teams regarding their work organization across site boundaries as well as regarding workforce representation	https://gute-agile-projektarbeit.de/	04/17–12/20	y
EdA	Empowerment in the digital world of work	• Development, testing and evaluation of sustainable design concepts for the transition to digital transformation in pilot projects<br>• development and application of a model based on a reciprocal relationship between self-responsible employees and organizational conditions making appropriate empowerment possible<br>• seven central design fields: agile organizational concepts, new management cultures, health promotion, time sovereignty, team empowerment, participation & co-determination, and crowd<br>• design concepts for strengthening empowerment in the digital working world are prepared for a broad range of users	http://eda-projekt.de/	01/17–12/20	y

eLLa4.0	sociodigital transformation towards excellent leadership and labor	• building up interaction competence and enabling managers of socio-digital systems by developing qualification modules for different target groups<br>• a training program "Excellent Socio-Digital Leadership" is developed and new learning worlds for the qualification of executives are being tested	https://ellaviernull.de/	09/19–08/22	y
Hierda	Humanization of digital work by Cowork-Spaces	• Development and testing of an innovative modularized organization, communication, and conflict management concept with various instruments for free and self-determined work in coworking spaces<br>• Distribution of the modular system and guidelines for good practice in coworking spaces in digital and printed form.	http://www.hierda.net	04/17–12/20	n
MegA [2]	Measures and recommendations for tomorrow's healthy work	• Development of a demographically sensitive HR and health management using conceptual and empirical research<br>• Development of an integrative, prevention-oriented modular multiplier system especially addressed to SMEs	http://www.gesundearbeit-mega.de	11/15–03/21	n
Praegewelt	Prevention-oriented design of the new world of work	• Development of new and healthy forms of work in the office<br>• Investigation of interactions between work organization, work styles, corporate culture and individual behaviors in different Open Space constellations<br>• Based on these results, working models for health-promoting open-space offices will be developed, implemented and subjected to a practical test with employees, managers and operational experts<br>• Different design options for individual and constellation-specific open-space offices are provided	http://www.praegewelt.de	01/16–07/19	n
PraeVierNull [2]	Prevention 4.0	• Development of concrete recommendations and guidelines for preventive work design in the world of work 4.0	http://www.praeventive-arbeit40.de	12/15–04/19	y
prentimo	Prevention-oriented design of mobile work	• Designing mobile work to be safe, healthy, appropriate to the different phases of life and gender-sensitive in order to reduce stress for employees and to strengthen the employees' loyalty to the company in the long term<br>• Four fields of action are being linked: mobility oriented work design, mobility-sensitive health promotion, preventive and reflective competence development and life-phase-oriented personnel management	http://www.prentimo.de	01/16–07/19	y
ReProNa	Reflexive project management for sustainable innovative work	• Development of management systems that produce salutogenic working and organizational conditions and that are constantly renewed under changing contextual conditions<br>• Structures and processes in project work are being redesigned<br>• Prevention is supposed to be based on the competence to design salutogenetic conditions in a contextually appropriate manner	http://reprona.de/	04/16–07/19	y

		• Project-specific burdens are analyzed, personal and organizational competences for coping with them are recorded<br>• Development of instruments for evaluating the sustainability of company work organization			
SANDRA	Designing the future world of work by availability management	• Development of solutions that improve the balance between work and private life, particularly regarding the accessibility of employees<br>• Organizational, legal and technical requirements for the introduction and commissioning of an operational availability management system are analyzed<br>• An availability manager was developed and tested with the company partners	https://www.sandra-projekt.de	04/17–06/20	y
SerWiss	Integrated design of business model and work for the international provision and commercialization of service-knowledge	• Development of an integrated solution providing comprehensive support for service and maintenance staff in carrying out the processes<br>• Technical (hardware and software) and business-oriented (business models and process design)<br>• The Knowledge-Centered Service (KCS®) methodology, integrating the development and maintenance of knowledge into the problem-solving process, is being used	https://www.produktion-dienstleistung-arbeit.de/de/projekte.php?PN = 11051562	03/20–02/23	n
SodA	Autonomy in companies of a digitized world of work	• Development and testing of models and measures in order to combine a holistic, socio-technically oriented implementation of digital technology with autonomy-promoting work and organizational design	http://www.soda-projekt.de/	06/17–05/20	n
teamIn	Digital leadership and technologies for tomorrow's interaction in teams	• Development of a mission statement for tomorrow's managers focusing on the growing demands of digitization<br>• Development of new management and communication tools as well as organizational structures and concepts to support managers in this process<br>• Creation of appropriate concepts for competence development	https://www.teamin-projekt.de	01/20–12/22	y
vLead	Resource-oriented and effective leadership models of digital project- and teamwork	• Development and testing of instruments for the resource-oriented and effective management of digital project and team work, especially for small and medium-sized enterprises (SMEs)<br>• Results are summarized and published in the so-called vLead Toolbox that supports managers in identifying the requirements of digital work, supporting team processes with information technology, designing meetings and innovation processes in a participatory manner and developing competencies for project work	http://www.vlead.de	04/17–12/20	y
WiViTe	Innovative knowledge spaces—transfer and generation of knowledge in virtual teams	• Investigation of how digital knowledge transfer and knowledge generation in virtual teams can be efficiently designed	https://www.produktion-dienstleistung-	02/19–01/22	y

	• Development and testing of a new method for work organisation based on a digital platform to support the creation, transfer and networking of knowledge in virtual collaboration	arbeit.de/de/projekte.php?PN = 11050913

[1] in part author's translation; [2] accompanying research connecting joint projects and pursuing research questions activities on their own; [3] based on the short descriptions provided in the database of the project management agency.
